# Metagenome and Metatranscriptome Analyses Using Protein Family Profiles

**DOI:** 10.1371/journal.pcbi.1004991

**Published:** 2016-07-11

**Authors:** Cuncong Zhong, Anna Edlund, Youngik Yang, Jeffrey S. McLean, Shibu Yooseph

**Affiliations:** 1 J. Craig Venter Institute, La Jolla, California, United States of America; 2 School of Dentistry, University of California Los Angeles, Los Angeles, California, United States of America; 3 Department of Periodontics, University of Washington, Seattle, Washington, United States of America; Stockholm University, SWEDEN

## Abstract

Analyses of metagenome data (MG) and metatranscriptome data (MT) are often challenged by a paucity of complete reference genome sequences and the uneven/low sequencing depth of the constituent organisms in the microbial community, which respectively limit the power of reference-based alignment and *de novo* sequence assembly. These limitations make accurate protein family classification and abundance estimation challenging, which in turn hamper downstream analyses such as abundance profiling of metabolic pathways, identification of differentially encoded/expressed genes, and *de novo* reconstruction of complete gene and protein sequences from the protein family of interest. The profile hidden Markov model (HMM) framework enables the construction of very useful probabilistic models for protein families that allow for accurate modeling of position specific matches, insertions, and deletions. We present a novel homology detection algorithm that integrates banded Viterbi algorithm for profile HMM parsing with an iterative simultaneous alignment and assembly computational framework. The algorithm searches a given profile HMM of a protein family against a database of fragmentary MG/MT sequencing data and simultaneously assembles complete or near-complete gene and protein sequences of the protein family. The resulting program, HMM-GRASPx, demonstrates superior performance in aligning and assembling homologs when benchmarked on both simulated marine MG and real human saliva MG datasets. On real supragingival plaque and stool MG datasets that were generated from healthy individuals, HMM-GRASPx accurately estimates the abundances of the antimicrobial resistance (AMR) gene families and enables accurate characterization of the resistome profiles of these microbial communities. For real human oral microbiome MT datasets, using the HMM-GRASPx estimated transcript abundances significantly improves detection of differentially expressed (DE) genes. Finally, HMM-GRASPx was used to reconstruct comprehensive sets of complete or near-complete protein and nucleotide sequences for the query protein families. HMM-GRASPx is freely available online from http://sourceforge.net/projects/hmm-graspx.

## Author Summary

Accurate analysis of microbial metabolism and function from metagenome and metatranscriptome data sets relies heavily on the comprehensive identification of protein family homologs present in these data. The task is routinely being done through alignment of the individual reads against the profile hidden Markov Models (HMM) of protein families in the reference database. This strategy, however, is hindered by the fact that the reads usually only represent partial protein sequences, which contain insufficient information for their accurate classification. To tackle this problem, we present a targeted assembly algorithm that, based on the sequence overlap information, simultaneously reconstructs complete or near-complete protein sequences and estimates their homology given the HMMs of the protein families of interest. The reconstructed protein sequences contain more complete information regarding the function of the corresponding protein, thus facilitating accurate annotation of themselves as well as the constituent sequencing reads. The resulting program, HMM-GRASPx, has been shown to have significantly improved performance (>40% higher recall rate with a similar level of precision rate) over other state-of-the-art counterparts such as RPS-BLAST and HMMER3.

This is a *PLOS Computational Biology* Methods paper.

## Introduction

Metagenomics (MG) and Metatranscriptomics (MT) are culture-independent methodologies [1,2] empowered by next-generation sequencing (NGS) technologies [3,4], which respectively enable genome and transcriptome profiling (RNA-seq) of the microbes in a given environment. MG studies allow the estimation of gene abundances to reconstruct the metabolic potential [5] as well as the taxonomic profile [6] of the microbial community. MT studies, on the other hand, can capture transcriptome-level changes of the microbial communities, possibly induced by longitudinal, cross-sectional environmental changes and/or intra- and inter-species interactions [7–10]. Identification of differentially expressed (DE) genes is based on comparisons of mRNA abundances across different conditions. Both MG and MT approaches rely heavily on accurate estimation of the DNA/mRNA abundances in the sample.

The abundance estimation in MG/MT data is traditionally solved through homology search, which aims at finding all homologs (as measured by sequence similarity) of the query reference. Given the reference sequence, individual NGS reads can be aligned against it using alignment programs such as Bowtie [11], BWA [12], BLAST [13], FASTM [14], RAPSearch [15,16], and DIAMOND [17]. Alternatively, the individual reads can be assembled into longer contig sequences using *de novo* assembly tools prior to the alignment [18,19]. Long contigs contain more complete structural features of the corresponding protein product and thus facilitate correct annotations. However, *de novo* assembly can be challenging due to uneven and/or low-coverage of the constituent organisms, leading to fragmentary assembly for many data sets. These issues have been partly alleviated through the *de novo* short peptide assembly approach [20,21] that aims at reconstructing complete protein sequences, and is not hampered by synonymous DNA mutations.

Previously, we developed a framework for identifying the homologs of a query protein sequence from a database of peptide reads that were translated from NGS reads (using fragmentary gene caller such as MetaGeneAnnotator [22] or FragGeneScan [23]). This framework, referred to as the simultaneous alignment and assembly (SAA) approach for short peptides, uses iterative alignment and assembly steps to improve homology detection, and integrates both the reference-based alignment and the targeted fragment assembly as a unified component [24,25]. It computes sequence similarity at each stage of contig extension, thus providing auxiliary sequence similarity information for guiding the graph traversal. Meanwhile, the alignments computed between the reference and the assembled contigs also more accurately reflect the true homology. Given the reference protein sequence, the algorithm attempts to recruit all of its homologous short peptide reads and assemble them into full-length proteins. This approach allows integration of the sequence overlapping information (i.e. between reads) with the sequence alignment information (i.e. between the read and the reference) while assessing homology. Intuitively, if a peptide read overlaps significantly with a second peptide read that aligns well with the reference protein sequence, it is more likely that the first read is also a homolog of the reference. The resulting program called GRASP (Guided Reference-based Assembly of Short Peptides) [24] and its computationally efficient version GRASPx [25] was shown to significantly improve sensitivity of homology search when compared to programs such as BLASTP and FASTM, and subsequently provide more accurate abundance estimation from MG/MT data. Currently, the SAA algorithm has only been implemented to accept a single sequence as the query. Motivated by improved homology detection capabilities of profile hidden Markov models (HMM), here we extend the SAA approach to enable the use of a profile HMM as a query.

Currently several methods are available for protein family profile-based homology search. HMMER3 [26–29] and RPS-BLAST [30,31] directly operate on individual reads and demonstrate low sensitivity [32,33]. To improve sensitivity, the Sensitive and Accurate protein domain cLassification Tool (SALT) [32] attempts to excessively recruit candidate homologous reads of the reference through a loose cutoff; then it applies *de novo* assembly on these recruited reads. Excessive recruitment of candidate homologs can have an impact on the running time of the program; the program is therefore only designed for analyzing single-genome data instead of large and complex MG/MT community data. SALT was later redesigned and extended to Xander [34], which was built on the spectrum alignment algorithm that was originally used for guided homology search on microarray data [35]. Xander also requires relatively large amount of computational resources to analyze tens or hundreds of protein families. On the other hand, the Ultrafast Protein Classification (UProC) toolbox [33] implements a computationally efficient approach to compare the MG/MT reads to the sequences of all members in the querying protein family (using the Pfam FULL alignment database), which makes it reference database-dependent. In summary, an accurate, efficient, and reference database-independent profile-based homology search program would greatly improve current MG/MT data analysis.

In this work we present a novel algorithm for profile HMM-based homology search that couples the banded Viterbi algorithm with the SAA framework. The resulting program HMM-GRASPx provides two important utilities. It can be used both as a homology search program that provides more accurate abundance estimation of the reference protein family, as well as a targeted assembly program that focuses on the reconstruction of genes sequences for the same protein family. Both of these features of HMM-GRASPx were benchmarked against HMMER3 [28], RPS-BLAST [30] and UProC [33]; and HMM-GRASPx demonstrated significantly improved performances. Moreover, its utility as a homology search program was further showcased using MG data sets from the Human Microbiome Project (HMP) [36] as well as MT data sets that were generated from *in vitro* grown oral biofilms [7]. HMM-GRASPx outperformed HMMER3 both in predicting the abundance of the antimicrobial resistance (AMR) gene families from the MG data set and in detecting DE genes that encode known biosynthesis pathways from the MT data set. In addition, HMM-GRASPx’s utility as a targeted assembly program was further demonstrated using the same *in vitro* oral biofilm MT data set. Targeted assembly using HMM-GRASPx predictions reconstructed more and longer contigs than using HMMER3 predictions, both in protein and nucleotide space. HMM-GRASPx is freely available online from http://sourceforge.net/projects/hmm-graspx.

## Results and Discussion

### The HMM-GRASPx design principle and analysis pipeline

Typical MG/MT data sets are generated from microbial communities whose compositions are not fully known *a priori* and often contain taxonomic groups that do not have representative reference sequences. In this case, the analysis of such MG/MT data sets involves homology search with available reference sequences that may be evolutionarily distant to some of the taxa in the sampled community. Computationally, the search for the remote homologs of a given query protein sequence is confounded by the combination of homologous and non-homologous protein sequences in the low sequence similarity alignment space [37]. The problem is further compounded by the current high-throughput sequencing technologies that generate short reads, which usually leads to short alignments that only contain partial information regarding the structural features of the protein. GRASP was designed to address these limitations by evaluating the sequence similarity between the query sequence and the reconstructed protein contigs, which resulted in longer alignments and improved accuracy [24]. HMM-GRASPx adopts a similar principle as GRASP for solving this problem, and further extends the utility to protein family profile-based homology search.

**Fig 1:**
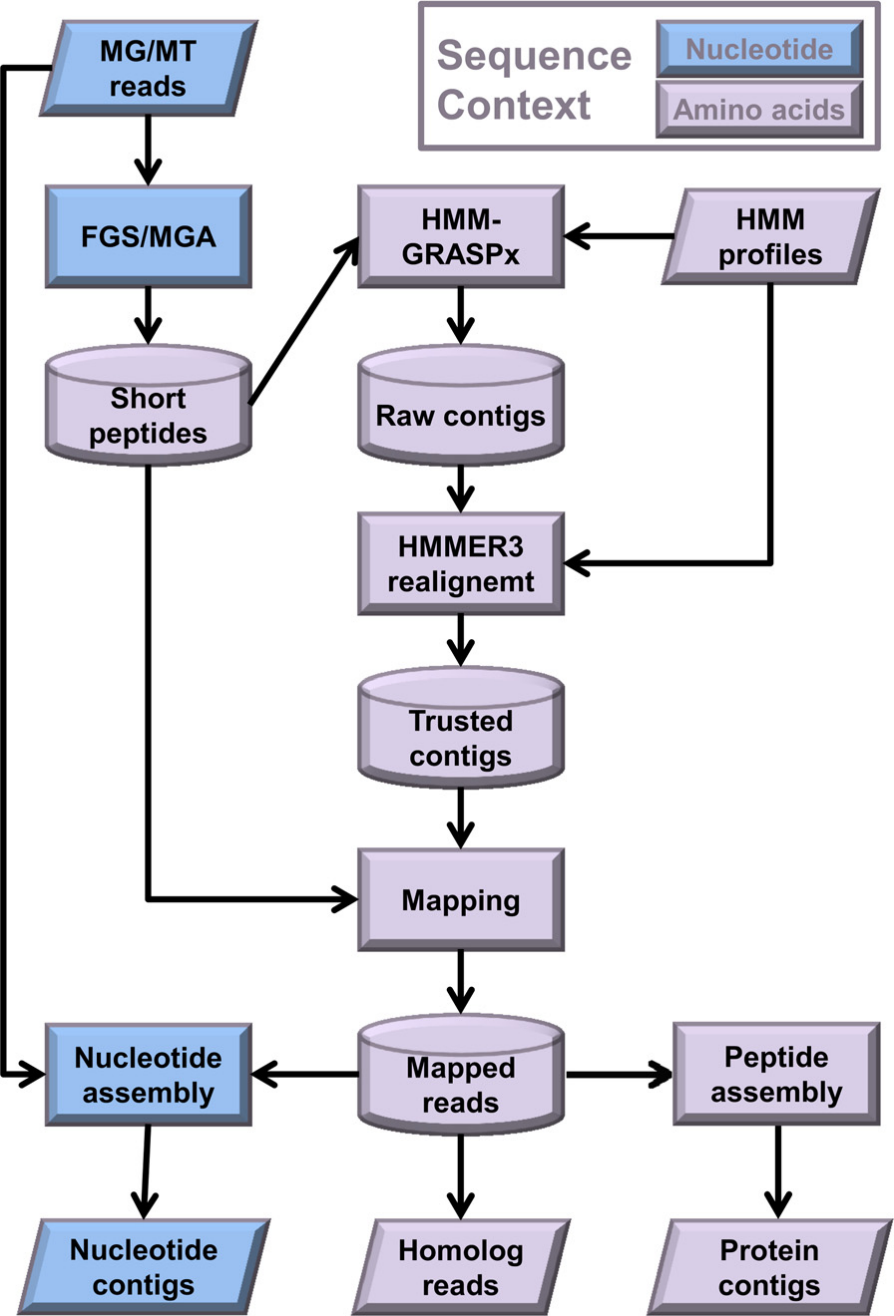
The HMM-GRASPx pipeline for homology search and gene-centric assembly. Blue shading of the objects indicates that the corresponding data or operation is in nucleotide space, while purple shading indicates amino-acid space.

The detailed algorithm for HMM-GRASPx is presented in the Methods section. An MG/MT data analysis pipeline using HMM-GRASPx as its core component is summarized in Fig 1 and includes the following steps.

- Partial protein coding sequences (referred to as *short peptide reads*) are identified from the raw MG/MT nucleotide reads using a gene-calling program (for instance, MetaGeneAnnotator [22] or FragGeneScan [23]). These short peptide reads, together with a query profile HMM, serve as the input to HMM-GRASPx.
- The SAA algorithm implemented in HMM-GRASPx uses the query profile HMM as a guide and operates on the short peptide reads database, assembling protein contigs that are subsequently verified through HMMER3 realignment.
- Homologous short peptide reads are recruited by mapping all reads to the verified contigs that are generated in the previous step.
- Targeted protein assembly can be performed using peptide assembler (e.g. SFA-SPA [21]) on HMM-GRASPx recruited short peptides.
- Targeted nucleotide assembly can be performed using nucleotide assembler (e.g. SPAdes [18]) on the original nucleotide sequences that correspond to the HMM-GRASPx recruited short peptides.

The “Mapping” step in Fig 1 corresponds to the homology search utility of HMM-GRASPx, while the “Nucleotide assembly” and “Protein assembly” steps in Fig 1 fully exploit its potential for targeted assembly. The current implementation of HMM-GRASPx outputs all possible traversals of its overlap graph as long as the corresponding contigs align well with the query, which may be redundant. Therefore, we recommend using other *de novo* assembly programs to further refine the set of output contigs (e.g. SPAdes [18] for nucleotide assembly and SFA-SPA [21] for protein assembly). In summary, the HMM-GRASPx based MG/MT analysis pipeline allows for estimation of gene/transcript abundances and targeted reconstruction of both protein and nucleotide sequences.

Asymptotic behavior for the running time of HMM-GRASPx is shown in S1 Fig. The expected running time of HMM-GRASPx grows linearly with the length of the sequences that are being assembled/searched.

### HMM-GRASPx accurately identifies homolog reads from simulated and real metagenomic data sets

HMM-GRASPx was benchmarked with homology search/classification programs including HMMER3 (version 3.1b2) [28], RPS-BLAST (version ncbi-blast+2.2.28) [30], and UProC (version 1.2.0) [33] on a simulated marine data set that contains 23 marine microbial genomes from the *Alteromonas*, *Candidatus*, *Erythrobacter*, *Flavobacteriales*, *Nitrosococcus*, *Photobacterium*, *Prochlorococcus*, *Roseobacter*, *Shewanella*, *Synechococcus*, and *Vibrio* groups (see Methods). The relative abundances of these bacteria were simulated according to their natural composition (coverages of each genome range from 1.25X to 10X with an average of 4X, see S1 Table). Three hundred and three Pfam protein families that are involved in important metabolic pathways were selected as the queries (S2 Table). Ground-truth homologous reads were defined by searching these protein families against the complete genomes and recruiting reads onto the identified homologous regions (see Methods). Given the ground-truth homologous reads, the recall rate, precision rate, and F-measure of the programs were calculated and compared (see Methods).

The performances of the four programs are summarized in Table 1. HMM-GRASPx shows the highest recall rate (~64.1% on average) among all programs. Both HMMER3 and RPS-BLAST show equivalently the highest precision rate (~94.3%); and HMM-GRASPx also shows reasonably high precision rate (~90.6%). Overall, HMM-GRASPx demonstrates the highest F-measure of ~75.0% on average, followed by RPS-BLAST which has an F-measure of ~47.4%. Furthermore, note that the increase of sequencing coverage would improve the assembly performance of HMM-GRASPx, which would in turn improve its overall performance. For example, when repeating the same experiment on a simulated data set in which all the 23 marine microbial genomes were *in silico* sequenced with an even 10X coverage, the average F-measure of HMM-GRASPx increased from ~75.0% to ~82.5%; the other programs were not benefited from the increased sequencing coverage (S3 Table).

**Table 1:** Performances of the programs for searching metabolic protein family profiles against the simulated marine data set with uneven coverage.

Pathway	#Families	HMM-GRASPx			HMMER3			RPS-BLAST			UProC		
		Rec.	Prec.	F.	Rec.	Prec.	F.	Rec.	Prec.	F.	Rec.	Prec.	F.
**KO00010**	47	**70.2**	94.3	**80.5**	25.0	95.7	39.7	43.8	**97.6**	60.4	26.8	68.0	38.5
**KO00020**	69	**61.6**	86.5	**72.0**	18.0	92.2	30.1	31.4	**94.5**	47.2	28.7	70.1	40.7
**KO00030**	21	**78.0**	96.2	**86.1**	28.6	**98.5**	44.4	49.2	97.7	65.5	33.1	56.4	41.7
**KO00051**	111	**58.7**	87.4	**70.2**	15.6	**94.1**	26.7	24.4	90.9	38.4	17.8	57.1	27.1
**KO00620**	80	**60.2**	89.4	**72.0**	15.3	92.9	26.3	27.1	**95.0**	42.2	23.6	75.6	35.9
**KO00680**	124	**59.3**	89.7	**71.4**	17.2	**95.1**	29.1	27.6	92.8	42.5	21.0	73.3	32.7
**KO00910**	49	**61.9**	87.3	**72.4**	16.3	**92.2**	27.7	29.5	88.8	44.2	26.7	76.9	39.6
**KO00920**	7	**62.7**	93.7	**75.1**	11.1	93.4	19.8	24.3	**97.0**	38.9	8.3	25.1	12.4
**Average**	-	**64.1**	90.6	**75.0**	18.4	**94.3**	30.5	32.2	**94.3**	47.4	23.2	62.8	33.6

Pathway names: KO00010 (Glycolysis/Glycogenesis), KO00020 (TCA cycle), KO00030 (Pentose phosphate pathway), KO00051 (Fructose and mannose metabolism), KO00620 (Pyruvate metabolism), KO00680 (Methane metabolism), KO00910 (Nitrogen metabolism), KO00920 (Sulfur metabolism). The column “#Families” indicates the number of protein (domain) families involved in the corresponding pathway. The columns “Rec.”, “Prec.”, and “F.” indicate Recall, Precision, and F-measure, respectively. All performances are presented as percentages. The highest performances among all programs are bolded.

Running time for all programs on the simulated marine data set is available in S4 Table.

### HMM-GRASPx accurately and completely reconstructs homolog protein sequences of query protein families

The utility of HMM-GRASPx as a protein family-specific targeted assembly program was evaluated on a real human saliva data set SRS013942 together with HMMER3, RPS-BLAST and UProC. A list of secondary metabolite synthesizing protein families collected from the antiSMASH2.0 database [38] were used as queries (S5 Table). The *de novo* peptide assembly program SFA-SPA [21] was used to assemble the homolog reads recruited by each of the programs (see Methods). The correctness of these assembled contigs was evaluated by re-aligning them against the querying profiles (see Methods), and we correspondingly define the true contigs (*t*.*c*.), true reads (*t*.*r*.), contig-level precision (*c*.*P*.), and the read-level precision (*r*.*P*.) (see Methods).

The performances of HMM-GRASPx, RPS-BLAST, and UProC are summarized in Table 2. HMMER3 was excluded from the precision benchmark because the program was used to define the ground-truth and will consistently have precision close to 100% (for number of true reads and true contigs for HMMER3 see S6 Table). HMMER-GRASPx recruited as much as two times and ~25% more true homologous reads compared to RPS-BLAST and UProC, respectively. Using RPS-BLAST predictions, SFA-SPA was able to assemble more true homologous contigs. However, the lengths of the RPS-BLAST contigs were considerably shorter than the ones assembled from HMM-GRASPx predictions (as measured by N50, see S7 Table). Aside from HMM-GRASPx’s higher sensitivity, the majority of its predicted reads were assembled into true homologous contigs, which corresponds to 96.8% read-level and 84.7% contig-level precision on average. RPS-BLAST was the second best-performing program with averaged values of 62.6% and 54.5%, respectively. These results show that HMM-GRASPx is well suited to perform protein family-specific targeted assembly.

**Table 2:** Performances of the programs for searching biosynthetic protein family profiles against the human saliva data set SRS013942.

Name	#Pfams	HMM-GRASPx				RPS-BLAST				UProC			
		#t.r.	r.P.	#t.c.	c.P.	#t.r.	r.P.	#t.c.	c.P.	#t.r.	r.P.	#t.c.	c.P.
Bacteriocin	13	**436**	**99.8**	29	**96.7**	182	69.2	**35**	49.3	56	0.2	17	0.1
B. Lactone	1	6	**100**	2	**100**	**9**	13.0	**4**	16.0	2	0.0	1	0.0
H. Lactone	1	9	**81.8**	1	**50.0**	**15**	10.3	**4**	11.4	0	0.0	0	0.0
Lanti pep.	6	**131**	**100**	21	**100**	46	82.1	**22**	73.3	36	0.1	13	0.0
NRPS	3	**15,037**	**98.9**	479	86.5	6,125	98.7	**623**	**93.1**	13,786	32.8	475	2.4
Oligo sac.	3	**32,393**	**98.5**	1,315	**89.9**	15,463	**98.5**	**1,662**	94.4	27,232	48.7	1,372	7.6
PKS	1	**913**	**96.8**	**11**	**52.4**	48	23.8	10	18.5	0	0.0	0	0.0
Terpene	3	**1,483**	**95.9**	**126**	**86.9**	411	94.0	92	86.8	19	0.1	7	0.0
Thiopeptide	1	**642**	**100**	19	**100**	333	74.0	**33**	47.1	350	0.8	17	0.0

“#Pfams” indicates the number of Pfam families involved in the biosynthesis of the corresponding secondary metabolite. Abbreviations: “B. Lactone”: Butyrolactones; “H. Lactone”: Homoserine lactone; “Lanti pep.”: Lantipeptides; “Oligo sac.”: Oligosaccharide. “#t.r.”: number of true reads; “r.P.”: read-level precision (percentage); “#t.c.”: number of true contigs; “c.P.”: contig-level precision (percentage). The highest performances among all programs are bolded.

Running time for all programs on the simulated marine data set is available in S8 Table.

### Antibiotic resistance gene family profiling of metagenomic data

Here we demonstrate the utility of HMM-GRASPx for functional profiling of bacterial communities by studying Anti-Microbial Resistance (AMR) gene families’ abundance profiles in HMP MG data sets [36]. AMRs are globally distributed and exist in most organisms and environments due to the ubiquitous presence of microbes. AMR represents a natural bacterial defense mechanism but has become a serious problem in treating infectious diseases due to the rapid spread of resistance genes, which makes it possible for bacteria to evade treatment [39,40]. Knowledge of the resistome profile in an individual has potential applications for clinical treatment of bacterial infections.

**Fig 2:**
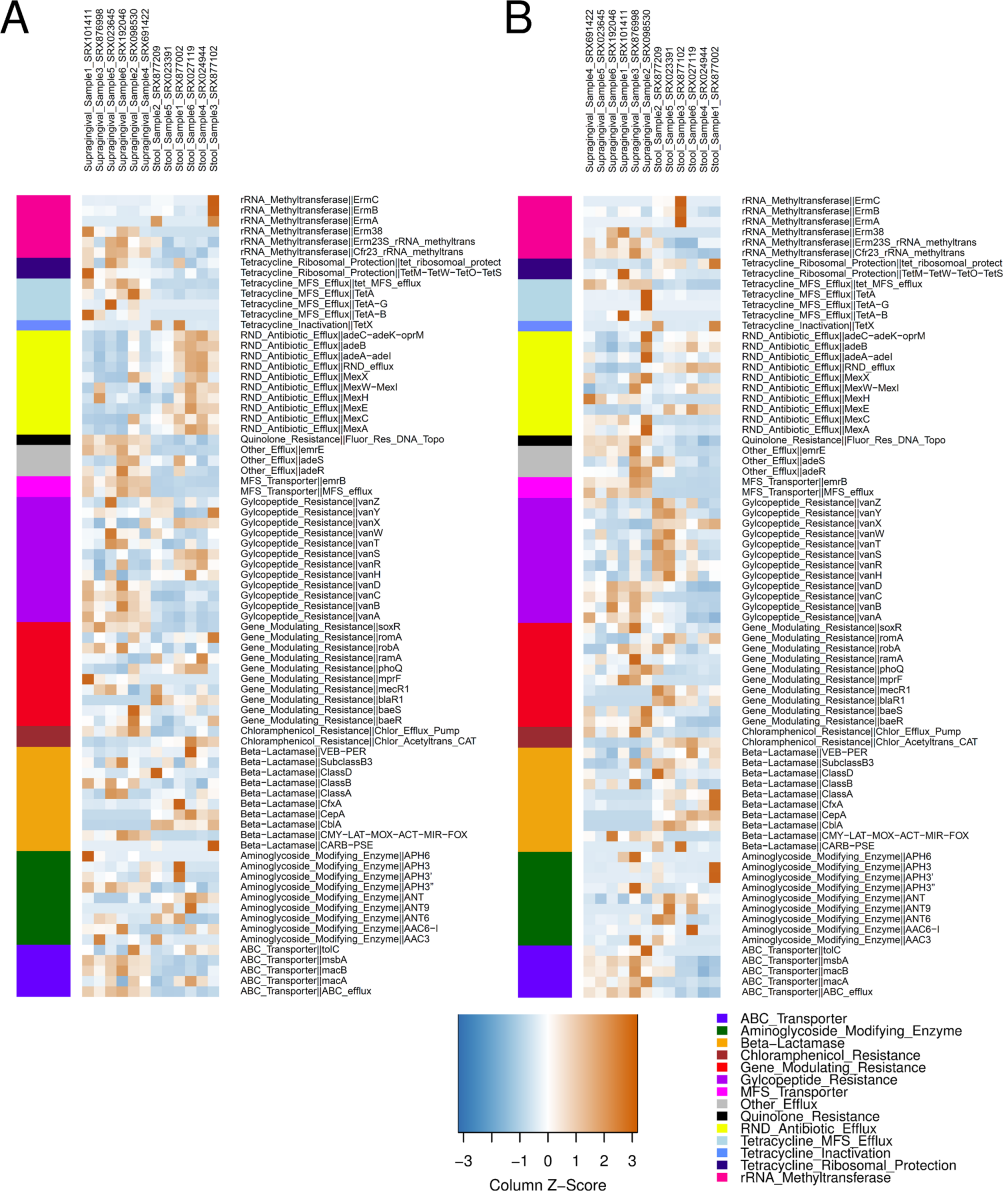
AMR protein-family profiles from the six supragingival and six stool samples as predicted by HMM-GRASPx and HMMER3. (A) AMR Profile predicted by HMM-GRASPx. (B) AMR Profile predicted by HMMER3. Hierarchical clustering was performed sample-wise (column-wise). Color bars on the left-hand-sides of the heat maps indicate AMR classification by RESFAM (bottom right legend). Abundance values (RPKM) were row-wise normalized into Z-scores (bottom right color key).

The AMR protein families registered in RESFAM [41] were selected as queries to profile the resistome (S9 Table). Twelve MG data representing six individuals, where each individual contributed to both a supragingival and a stool data set, were selected for this experiment (S10 Table). HMM-GRASPx and HMMER3 were individually applied to search the AMR profiles against these data sets (see Methods). Presented AMR protein families were selected (S9 Table) and their raw counts were further normalized into Reads Per Kilobase per Million (RPKM) to represent their abundance profile of the given MG data set (see Methods). The predicted AMR abundance profiles were further clustered using hierarchical clustering algorithm to show body site-specific resistome (see Methods). The clustering results of HMM-GRASPx- and HMMER3-predicted profiles are shown in Fig 2A and 2B, respectively.

The results indicate that different body sites harbor bacterial communities containing distinct resistome profiles, which is in line with previous findings of the human microbiome where distinct biogeographical signatures were observed for bacterial species at 22 different body sites (including stool and oral plaque) [42]. For example, a clear difference between body sites was revealed by both programs for several genes involved in vancomycin resistance (i.e. D-Ala-D-Ala ligase genes *vanA*, *vanB*, *vanC*, and *vanD*) (Fig 2A and 2B, see Glycopeptide_Resistance category), which could only be identified in MG libraries obtained from oral supragingival plaque and not from the stool samples. This difference may be due to that vancomycin targets Gram-positive bacteria (the outer membrane of most Gram-negative bacteria is impermeable to large glycopeptide molecules such as vancomycin), which are highly abundant in the oral plaque and less abundant in stool. On the other hand, while the biological environment does show a stronger impact in shaping the resistome, personalized difference can indeed be observed for several AMR protein families. For example, both programs predicted high abundance of *ErmA*, *ErmB*, and *ErmC* genes specifically in the stool sample SRX877102 (Fig 2A and 2B, see rRNA_Methyltransferace category). The observation of personalized resistome profile may be explained by the antibiotics exposure history of the specific individual [43], and can be referred to when designing personalized treatment plans.

Although both HMM-GRASPx and HMMER3 made similar predictions on abundances of the AMR protein families for most of the cases, a notable difference is that HMM-GRASPx predicted a high abundance of all ten resistance-nodulation-division (RND) antibiotic efflux gene families in stool samples, while the HMMER3 predicted abundance profiles for these gene families are less consistent (five with higher abundances in supragingival plaque samples and the other five with higher abundances in stool samples). It is clear that HMM-GRASPx generated more biological meaningful predictions, because RND gene families are unique for Gram-negative bacteria (e.g. *Bacteroides*, which is abundant in healthy stool samples) and should be in low abundance in the Gram-positive rich healthy supragingival plaque samples. In addition, differences in abundance predictions further affect clustering of gene families based on the abundance profiles. Further gene family-wise WPGMA clustering algorithm clustered 7 out of the 10 RND gene families together using HMM-GRASPx predictions (S2 Fig), compared to only 2 out of 10 using HMMER3 predictions (S3 Fig). These results suggest that HMM-GRASPx improves accuracy of functional characterization and profiling for MG data sets.

### HMM-GRASPx improves differential expression analysis for metatranscriptomic data

MT data is routinely generated to profile the transcription level of genes, and comparison of MT data sets across different conditions and/or time points can be used to identify DE genes. Such information can be used to investigate which genes and metabolic pathways are critical in responding to environmental disturbances and how microbial communities adapt to environmental changes. The majority of the MT annotation analysis workflows rely heavily on available reference genomes, which serve as templates for read mapping. Other transcriptome assembly approaches are hampered by a similar issue of low/uneven coverage of the expressed transcripts by the microbial community. MT analysis of unexplored environments with high microbial-community complexity and very few genome sequence representatives is still an open problem.

To demonstrate both the utility of HMM-GRASPx for protein family-level MT analysis and how its improved homology search performance also improve DE analysis, we selected eight human oral biofilm MT data sets generated from a taxonomically well-defined oral *in vitro* biofilm model system [7,44]. These data sets were generated to study health-associated molecular processes, which contribute to protection from caries diseases (tooth enamel mineralization). Gene transcription responses were monitored in a temporal manner while the oral *in vitro* biofilm community metabolized sugar which resulted in a classic pH drop of the growth media followed by a health-associated pH recovery [7]. The MT data sets (SRA: SRP049210) contain replicate libraries obtained from biofilm samples collected at 0 hour (pH 7.0, three replicates), 6 hours (pH 4.2, two replicates), and 9 hours (pH 5.2, three replicates) after glucose amendment (S11 Table). Since very little knowledge exists on the role of bacterial interactions and cell-to-cell signaling (i.e. antagonist or cooperative interactions) in human oral health, we focused on the identification of biosynthetic protein families that potentially synthesize signaling molecules. The biosynthetic protein families were selected from the antiSMASH2.0 database [38] (S12 Table). HMM-GRASPx and HMMER3 were applied individually to search all MT libraries (see Methods).

**Fig 3:**
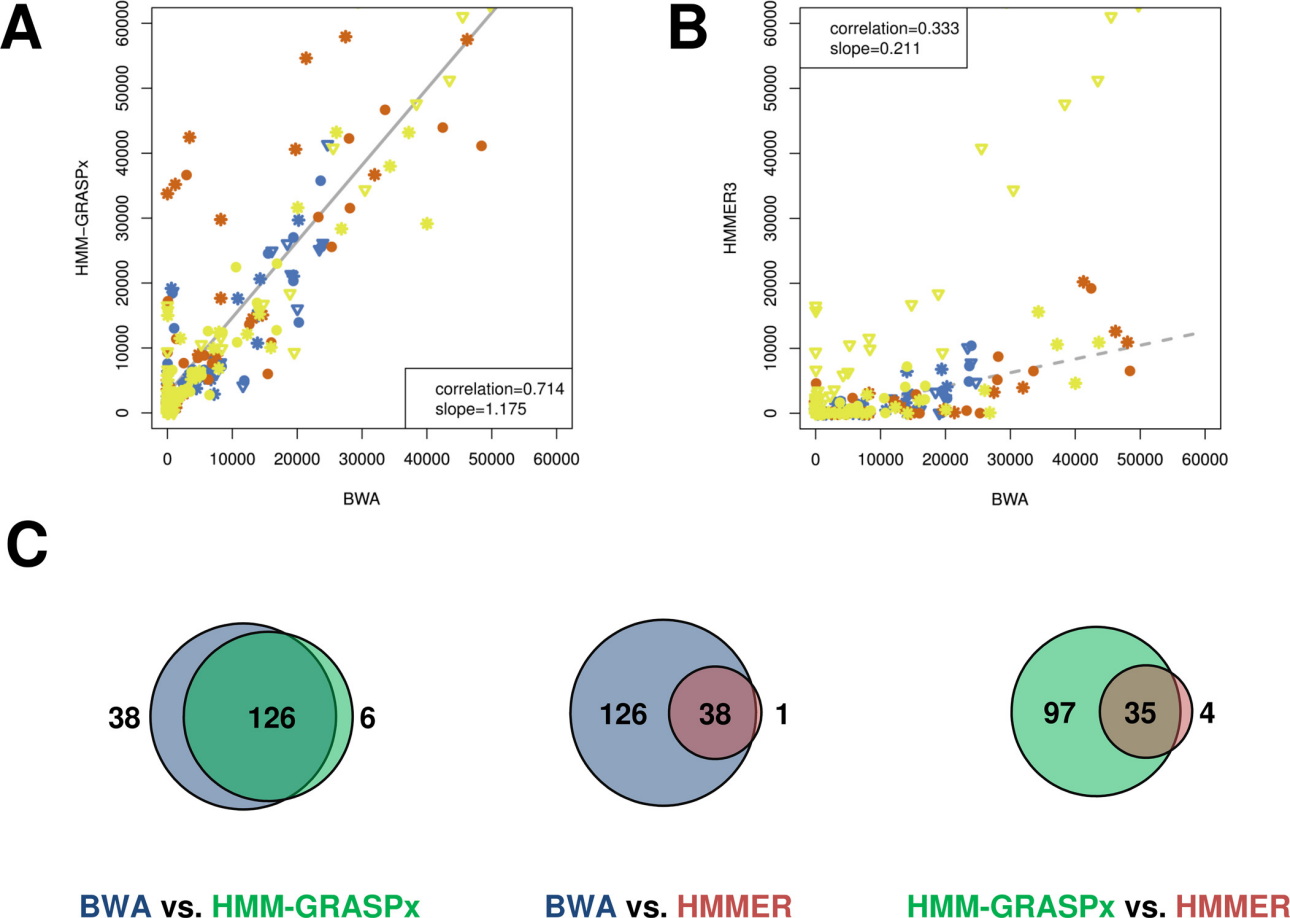
Comparison of HMM-GRASPx- and HMMER3-generated results from the *in vitro* human oral plaque biofilm MT data set. (A) Abundances correlation between HMM-GRASPx predictions and the ground-truth (i.e. BWA). The x- and y-axis indicate the number of homologous reads recruited by the corresponding programs. Colors indicate different conditions where the libraries were constructed (blue for 0hr/pH 7.0, red for 6hr/pH 4.2, and yellow for 9hr/pH 5.2). Different marks indicate different replicates. (B) Abundances correlation between HMMER3 predictions and the ground-truth. (C) Venn diagram showing the overlap between detected DE genes using HMM-GRASPx predictions (green), HMMER3 predictions (red), and the ground-truth homologous reads (blue).

Previous analysis of the MT data sets showed that 14 key bacterial species were highly active across different pH stages [7]. These species belong to the *Streptococcus*, *Lactobacillus*, *Fusobacterium*, *Gemella*, *Klebsiella*, and *Veillonella* genera. The reference sequences of these 14 strains were used to construct the ground-truth set for benchmarking purpose (see Methods). Benchmark results are summarized in Table 3, which shows that HMM-GRASPx has a higher recall rate (~80% compared to ~15%) but a lower precision rate (~80% compared to ~90%) than HMMER3. The results are consistent with the previous benchmark results on the simulated marine data set. Overall, HMM-GRASPx showed >50% higher F-measure. Spearman’s correlation between the predicted and the ground-truth abundance was calculated for both HMM-GRASPx (Fig 3A) and HMMER3 (Fig 3B). The abundances predicted by HMM-GRASPx were more consistent (Spearman’s correlation 0.714) to the ground-truth abundances than HMMER3 (Spearman’s correlation 0.333). Linear regression analysis also showed that HMM-GRASPx over-predicted the abundance by 18% (slope 1.18), while HMMER3 underestimated the true abundances by 79% (slope 0.21).

**Table 3:** Performance of HMM-GRASPx and HMMER3 for searching biosynthetic protein family profiles against the *in vitro* human oral plaque biofilm MT data sets.

Sample	#Reads	HMM-GRASPx			HMMER3		
		Recall	Precision	F-measure	Recall	Precision	F-measure
**0H.1**	22,280,139	**81.5**	83.0	**82.3**	15.8	**90.5**	26.9
**0H.2**	17,687,166	**81.9**	88.4	**85.1**	16.4	**91.6**	27.8
**0H.3**	23,106,973	**82.4**	88.0	**85.1**	16.0	**91.6**	27.3
**6H.1**	31,229,289	**81.0**	74.2	**77.4**	16.2	**90.4**	27.5
**6H.2**	43,702,127	**83.2**	78.5	**80.8**	16.7	**95.0**	28.4
**9H.1**	15,550,857	**79.2**	84.1	**81.6**	16.7	**91.8**	28.2
**9H.2**	37,336,599	**82.6**	86.8	**84.6**	14.5	**93.2**	25.1
**9H.3**	42,394,221	**81.9**	77.3	**79.5**	14.5	**91.4**	25.1

The gene-expression underestimation by HMMER3 represents a reduced statistical power in downstream DE analysis, which subsequently leads to false negative predictions. The abundances predicted by HMM-GRASPx and HMMER3 were used to identify the DE genes in the 14 key species of the community using the DESeq2 program [45] (see Methods). Fig 3C shows that the majority of the true DE genes were successfully identified using HMM-GRASPx predictions, resulting in a Jaccard similarity of 0.74 with the ground-truth. On the other hand, HMMER3-based DE gene detection only showed a Jaccard similarity of 0.23. The majority (89.7%) of the HMMER3-based DE genes were contained in the set of HMM-GRASPx-based DE genes. The results suggest that the improved homology search accuracy brought by HMM-GRASPx will also improve DE gene analysis.

### HMM-GRASPx improves targeted assembly of metatranscriptomic data

The above benchmark for strain-level DE analysis was made possible through using the reference sequences of the 14 core genomes to partition the recruited homolog reads. In practice, reference genomes may not always be available, especially for less-explored environments. In this case, it might be challenging to obtain strain-level expression of the protein families from direct application of homology search programs such as HMM-GRASPx, HMMER3, or RPS-BLAST. A potential solution to this problem is to first assemble the recruited homologous reads (Fig 1, “Peptide assembly” and “Nucleotide assembly”) followed by taxonomic binning of the resulting contigs [46,47]. DE analysis within individual taxonomic bins would allow for monitoring of transcription activities of different taxonomic groups among environmental changes.

Here, using the *in vitro* oral biofilm MT data sets, we benchmarked the qualities of the assemblies that were generated by using HMM-GRASPx- and HMMER3-recruited reads. Protein families in the antiSMASH2.0 database [38] that are longer than 200 HMM states were used as the queries for this experiment (S12 Table). Peptide assembly was performed using SFA-SPA [21]; and nucleotide assembly was performed using SPAdes [18] (see Methods). Only peptide contigs that are longer than 60aa and nucleotide contigs that are longer than 180nt were considered (see Methods). Refer to the targeted assembly of a query protein family on a MT data set as an *assembly case*.

**Fig 4:**
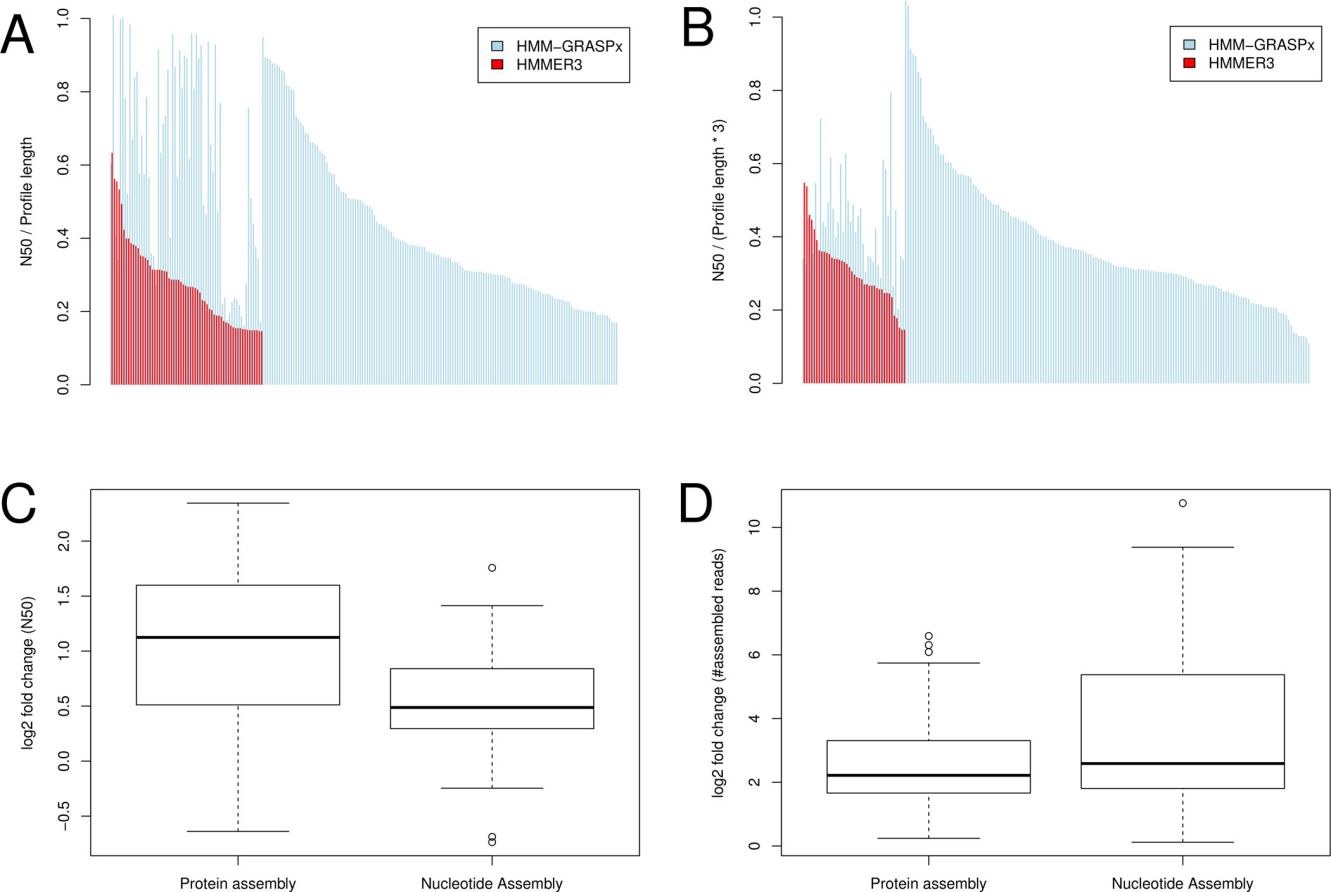
Targeted assembly of secondary metabolite synthesizing protein families from the *in vitro* human oral biofilm MT data set. Only the protein contigs longer than 60aa and nucleotide contigs longer than 180nt were considered. (A) Normalized N50 for protein assembly. (B) Normalized N50 for nucleotide assembly. For (A) and (B), red color indicates the performance of HMMER3 and blue color indicates HMM-GRASPx. The x-axes indicate assembly cases and were sorted based on the decreasing values of the HMMER3 performance and then the decreasing values of the HMM-GRASPx performance. Assembly cases without corresponding red bars indicate that no contig was assembled using HMMER3 predictions. (C) Log2 fold change for the N50 measures in assembly cases where contigs can be assembled using either HMMER3 or HMM-GRASPx prediction. (D) Log2 fold change for the number of assembled reads in assembly cases where contigs can be assembled using either HMMER3 or HMM-GRASPx prediction.

For protein assembly, SFA-SPA was able to assemble more and longer contigs using HMM-GRASPx predicted reads. Concretely, SFA-SPA generated at least one contig for 227 assembly cases using HMM-GRASPx-predicted reads (blue bars in Fig 4A), while it generated at least one contig for only 64 assembly cases using HMMER3-predicted reads (red bars in Fig 4A). Fig 4A also shows that for the overlapping cases between HMM-GRASPx-based assembly and HMMER3 based assembly (totaling 63), HMM-GRASPx-based contigs have higher normalized N50 (see Methods) than HMMER3-based contigs (in Fig 4A where blue and red bars overlap, blue bars are generally higher than red bars). Fig 4C shows that on average, the normalized N50 for HMM-GRASPx-based peptide contigs is 2.3 fold higher than HMMER3-based peptide contigs. Fig 4D also shows that HMM-GRASPx-based peptide assembly recruited 18.3 fold more reads than HMMER3-based peptide assembly. We subsequently assess the precision of the assembled contigs by realigning them back to the query HMM using HMMER3. Among the 1,263 HMM-GRASPx-based contigs, 1,154 can be aligned to the corresponding query HMM (precision 91.4%, see Methods). As expected, all 279 HMMER3-based contigs were successfully aligned (due to that HMMER3 was used in both the search and verification steps).

For targeted nucleotide assembly, the same trend that HMM-GRASPx-based assembly generated more and longer contigs and maintains high precision was also observed. Specifically, Fig 4B shows that SPAdes [18] generated at least one contig for 220 assembly cases using HMM-GRASPx-predicted reads (blue bars), while it generated at least one contig for only 41 assembly cases using HMMER3-predicted reads (red bars). Fig 4B also shows that the normalized N50 measures of the HMM-GRASPx-based contigs are generally higher than those of HMMER3-based contigs for the overlapping 41 assembly cases. Fig 4C shows that on average, the normalized N50 for HMM-GRASPx-based peptide contigs is 1.5 fold higher than HMMER3-based nucleotide contigs. Fig 4D also shows that HMM-GRASPx-based peptide assembly recruited 91.8 fold more reads than HMMER3-based nucleotide assembly. Among the 3,842 HMM-GRASPx-based contigs, 3,437 of them were successfully verified with E-value less than 0.01 (precision 89.4%). All 412 HMMER3 based contigs were successfully verified.

For the 3,437 verified nucleotide contigs, we further evaluated their taxonomic origins by searching these sequences using BLASTN against the NT (non-redundant nucleotide) and using BLASTX against the NR (non-redundant protein) databases (see Methods). BLASTX was able to identify high-scoring hits from the NR database for all 3,437 nucleotide contigs, while BLASTN failed to align 507 (14.8%) of them against the NT database. We also attempted to use KRAKEN [48] with the entire RefSeq as the reference database to annotate these 507 contigs but failed on 506 or 99.8% of them (see Methods). A potential reason for not finding these contigs’ homologs from the NT database is that they might harbor a significant amount of neutral mutations and originate from previously uncharacterized microbial species. The BLASTX search results for these contigs can be used to infer the relationship between these uncharacterized microbial species and known bacterial species (S4 Fig). In additional to taxonomic inference, the nucleotide sequences assembled using HMM-GRASPx can also provide valuable information for further design experiments (e.g. PCR primer design) for evaluating the expression levels or investigating the functions of the protein families.

### Conclusions

In summary, we developed a novel profile-based SAA algorithm and implemented the idea into the HMM-GRASPx program. HMM-GRASPx aims at highly accurate MG/MT data analysis in the absence of complete reference databases. Here we demonstrate that HMM-GRASPx significantly improves homology search and facilitates functional profiling of MG data sets for accurate and robust characterization and comparison of microbial communities. The program was also shown to improve DE detection in MT data sets, which will lead to improved identification of metabolic pathways and coverage of genes within these pathways that are under differential regulation. Finally, HMM-GRASPx can also be used for targeted assembly, which can subsequently enable a more in-depth analysis of taxonomy and protein family function. The program HMM-GRASPx is freely available online from http://sourceforge.net/projects/hmm-graspx.

## Methods

### HMM-GRASPx algorithm

The core algorithm of HMM-GRASPx was developed as an integration of the banded Viterbi algorithm for HMM parsing and the GRASPx SAA engine that iteratively computes sequence similarity and extends contigs based on overlapping short peptide sequences in the database. The seeding and the alignment modules are substantially different from GRASPx; and we focus on describing them here. Details for the other modules can be found in Zhong *et al*. [25].

HMM-GRASPx begins with seeding, a process that aims at anchoring the initial alignment based on highly similar regions shared between the reference and the target. When searching an HMM against the short-peptide database, seeding is performed by identifying high-scores matchings *k*-grams (*k*-gram refers to *k* consecutive match states in the reference HMM model or *k* consecutive amino acids in the target). Define the *maximum seed score* for the *k*-grams in the reference as the highest log odds-ratio for any sequence that can be emitted from the *k*-grams. A score scale (between 0 and 1) can be applied to the maximum seed score to define a position-specific cutoff for seeding. HMM-GRASPx further incorporates an efficient seeding heuristic through adopting the idea of reduced-alphabet [15] for pre-filtering target *k*-grams.

The alignment component of HMM-GRASPx adopts the banded Viterbi algorithm. The Viterbi algorithm computes the most probable parse of a sequence given the HMM. The dynamic programming algorithm can be summarized as the following recursive functions (for extension to the C-terminus; extension to the N terminus can be derived analogously).

$$M[i,j]=emit(M_{i},j)+\text{max}\left\{ \begin{array}{l} M[i-1,j-1]+tran(M_{i-1},M_{i})\\ I[i-1,j-1]+tran(I_{i-1},M_{i})\\ D[i-1,j]+tran(D_{i-1},M_{i}) \end{array}\right.\;,$$

$$I[i,j]=emit(I_{i},j)+\text{max}\left\{ \begin{array}{l} M[i,j-1]+tran(M_{i},I_{i})\\ I[i,j-1]+tran(I_{i},I_{i}) \end{array}\right.\;,$$

$$D[i,j]=\text{max}\left\{ \begin{array}{l} M[i-1,j]+tran(M_{i-1},D_{i})\\ D[i-1,j]+tran(D_{i-1},D_{i}) \end{array}\right.\;.$$

The first index *i* refers to the reference and the second index *j* refers to target. The tables *M*, *I*, and *D* store scores (log odds-ratio) for the most-likely parse, provided that the current-stage parse ends in the match, insert, or delete states of the HMM, respectively. The emission probabilities *emit* and the transition probabilities *trans* are coded in the HMM models. The banding was implemented through only computing table entries which satisfy the condition |*i* − *j*| ≤ *d*, where *d* is the band size.

The limitations of the above Viterbi algorithm are two-fold. First, the seeding and banding constraints violate the compositional assumption (i.e. the probabilities of all possible sequences no longer add up to 1 for the given parameters); the resulting statistics should only be used for ranking the targets. Second, the Viterbi algorithm only computes the best parse of the sequence, while the Forward-backward algorithm considers all parses and provides more meaningful statistical measures [49]. To avoid both limitations, HMMER3 is used re-compute the scores for HMM-GRASPx assembled protein contigs. The overhead of this step is marginal compared to the main assembly module.

The HMM-GRASPx algorithm can be summarized in the following steps:

1. Indexing: HMM-GRASPx constructs the extension links for the given MG/MT database, which is conceptually analogous to the overlap graph [25].
2. Seeding: The seeds are identified through identifying high-score *k*-grams between the reference and the target. The reads that contain the corresponding *k*-grams are used as initial contigs for further extension.
3. Extension: HMM-GRASPx extends the current contigs based on the pre-constructed extension links that are associated with the contigs’ terminating reads. For more details please refer to [25].
4. Alignment: The alignment between the querying HMM and the current contig is evaluated using the banded Viterbi’s algorithm described above. HMM-GRASPx at this step decides whether to continue using both log odds-ratio drop-off and read redundancy [25]. If it decides to continue, the algorithm loops back to the previous Extension step.
5. Recalibration: After all initial contigs being extended, the algorithm attempts to further assemble this contigs to increase their lengths. For more details please refer to [25].
6. Realignment: The recalibrated contigs are realigned with the querying profile HMM to recalculate the statistics using HMMER3.
7. Mapping: Short peptides are subsequently mapped against the trusted contigs.

### Benchmark experiment on the simulated marine metagenomic data set

Twenty three bacterial genomes were chosen for the simulation as they were found prevalent in marine environments [50] (S1 Table). According to their natural compositions, each genome was *in silico* sequenced with different coverage (min coverage 1.25X, max coverage 10X, mean coverage 4X). WGSIM (version 0.3.0) was used for *in silico* sequencing to generate pair-end reads with error rate 0.01 and expected read length 100; and the number of reads to be generated were calculated using the genome sizes and the expected coverages (parameter for running WGSIM: ‘-e 0.01–1 100–2 100 -N [num_reads]’). The nucleotide reads were then translated into short peptide reads using FragGeneScan (version 1.17) [23] with parameters ‘-complete = 0 -train = illumina_10’. The resulting simulated data set is available online from https://sourceforge.net/projects/hmm-graspx/files/SupplementaryData/SimMarine_23G.fa.tgz.

A set of Pfam (version 27.0) protein families which involve in important metabolic pathways (protein family-pathway association was made using the Kyoto Encyclopedia of Genes and Genomes, or KEGG, which was obtained in Jan. 2015) was used as queries for the benchmark experiment (S2 Table). The ground-truth homolog reads for a given protein family was defined by searching the protein family profile against the complete genomes. Concretely, the protein family profile HMM was searched against all proteins encoded (coding regions were extracted from KEGG) by the 23 genomes listed in S1 Table using HMMER3 with default parameters. All identified regions that are passing the HMMER3 (version 3.1b2) trusted-cutoff (i.e. E-value 0.01) were selected as homologous regions. Such a setting was applied to all HMMER3 runs referred in this work. Note that one protein sequence may contain multiple homolog regions with each of them assigned to different Pfam families; this is because one protein sequence may contain multiple domains. However, each region can be assigned to only one protein family; if the region was able to be aligned to multiple protein families, it would be assigned to the protein family that generates the most significant E-value. The simulated peptide reads were then mapped against these homologous regions using an in-house short-peptide mapper ‘graspx-map’ (with parameters ‘—num_errors = 3—portion_mapped = 0.6’, which requires that a short peptide be mapped to the reference for >60% of its sequence with less than three substitution errors). Such a setting was applied to all ‘graspx-map’ runs referred in this work. The program ‘graspx-map’ is released together with the HMM-GRASPx software package. The ground-truth homologous reads are available online from https://sourceforge.net/projects/hmm-graspx/files/SupplementaryData/SimMarineGroundTruth.tgz.

The performance of each homology search program was measured as follows. Define true positives (TP) as the successfully predicted homologous reads, false positives (FP) as the predicted non-homologous reads, and false negatives (FN) as the un-predicted homologous reads. Subsequently, define Recall rate and Precision rate as:

$$\text{Re}call=\frac{TP}{TP+FN},\;\text{Pr}ecision=\frac{TP}{TP+FP}.$$

The F-measure, a weighted average of recall and precision, was then defined as

$$F=\frac{2*\;\text{Re}\;call\;*\;\text{Pr}\;ecision}{\text{Re}\;call+\text{Pr}\;ecision}.$$

The profile HMMs of the selected protein families were retrieved from of Pfam version 27.0. The corresponding position-specific scoring matrix (PSSM) profiles (required by RPS-BLAST as inputs) for these protein families were retrieved from the Conserved Domain Database (CDD, downloaded on Oct. 24^th^, 2014) [30]. HMM-GRASPx was run in its default mode. Default parameters of HMM-GRASPx are detailed as follows. The seed length for alignment initialization is set to 6 (in reduced alphabet GBMR4, see definition in [15]); the minimum overlap length for assembly is set to 10; the alignment band-size is set to 20; the maximum assembly depth is set to 5; the HMM-GRASPx *P*-value cutoff was set to 0.05; and the HMMER3 verification E-value cutoff was set to 0.01. Such a setting was applied to all HMM-GRASPx runs referred in this work. Read recruitment was performed using ‘graspx-map’. HMMER3 [28] was run with its default parameters. RPS-BLAST (version ncbi-blast+2.2.28) [30] was run with its default parameters except that its E-value cutoff was set to 0.001 (‘-evalue 0.001’). We tuned this parameter so as to match the precision rate of RPS-BLAST with the other programs (~90%). Such a setting was applied to all RPS-BLAST runs referred in this work. UProC (version 1.2.0) [33] was run using its default parameters with Pfam seed alignments as the reference database. We chose Pfam seed alignments instead of Pfam full alignments for fair comparison; because the query profiles of the other programs, i.e. HMM-GRASPx, HMMER3, and RPS-BLAST, were built from Pfam seed alignments.

### Benchmark experiment on the human saliva metagenomic data set SRS013942

The data set was first retrieved from NCBI Sequence Read Archive (SRA) using the accession ID SRS013942 and then quality trimmed using CLC Assembly Cell (version 4.0.12) using parameters ‘-f 33 -m 55’, which only keeps reads that are longer than 55bp after the trimming. Low-complexity regions of the reads were detected using DUST (from ncbi-blast+2.2.28) with default parameters; reads with >40% of their sequences being masked were subsequently discarded. The preprocessed nucleotide reads were then translated into peptide reads using FragGeneScan (version 1.17) [23] with parameters ‘-complete = 0 -train = illumina_10’. The data set contains 12,036,685 reads in total and is available online from FragGeneScan-called peptide reads available from https://sourceforge.net/projects/hmm-graspx/files/SupplementaryData/Saliva.faa.tgz.

A list of protein family profiles compiled from the antiSMASH2.0 database (downloaded on May 29^th^, 2015) was used as the queries (S5 Table). Because RPS-BLAST does not build PSSMs out of the multiple alignments from scratch, only the Pfam profiles in the antiSMASH2.0 database were used as the queries for this exercise because their corresponding PSSMs can be directly retrieved from CDD. HMM-GRASPx, HMMER3, and RPS-BLAST were run with the parameters described in the previous section. Note that UProC was allowed to use Pfam full alignments as its reference database as recommended by the UProC authors to optimize its performance. The reads recruited by each of the programs were then assembled using SFA-SPA (version 0.2.1) [21] using default parameters. The resulting assembled contigs are available online at https://sourceforge.net/projects/hmm-graspx/files/SupplementaryData/SalivaAssembledContigs.faa.tgz. True contigs (*t*.*c*.) were defined as the contigs that can be aligned with the querying protein family profile using HMMER3 and passing the trusted E-value cutoff 0.01; true homologous reads (*t*.*r*.) were defined as the reads that can be mapped onto the true contigs using ‘graspx-map’. Correspondingly, the read-level (*r*.*P*.) and contig-level (*c*.*P*.) precision rate for each programs were respectively defined as follows:

$$r.P.=\frac{\#true\;\text{Re}\;ads}{\#total\;\text{Re}\;ads}\;\text{and}\;c.P.=\frac{\#trueContigs}{\#totalContigs}.$$

### Analysis of the human supragingival and stool metagenomic data sets

Twelve metagenomic data sets from six healthy individuals (each individual contributing to one supragingival and one stool data set) were downloaded from NCBI SRA using the accession numbers provided in S4 Table. Trimmomatic [51] was used to trim the reads with parameters ‘ILLUMINACLIP:TruSeq3-PE-2.fa:2:30:10 LEADING:3 TRAILING:3 SLIDINGWINDOW:4:15 MINLEN:75’. The trimmed nucleotide reads were then translated into peptide reads using FragGeneScan (version 1.17) [23] with parameters ‘-complete = 0 -train = illumina_10’.

A list of AMR gene families from RESFAM (downloaded on Aug. 12^th^, 2015) [41] was used as the query (see S5 Table). HMM-GRASPx and HMMER3 were run as previously described. Only the protein families that had at least 10 homolog reads predicted by both HMM-GRASPx and HMMER3 were selected for further analysis. In total 77 protein families were selected. The abundances of these 77 protein families were represented as the RPKM; and the RPKM data is available online from https://sourceforge.net/projects/hmm-graspx/files/SupplementaryData/RESFAMResults.tgz. Sample-wise hierarchical clustering was performed on the RPKMs using the R function ‘heatmap.2’ with one minus Spearman’s correlation as the distance measure and the Weighted Pair Group Method with Averaging (WPGMA) as the clustering algorithm (parameters used: ‘scale = "col", hclust = function(x) hclust(x,method = "mcquitty"), distfun = function(x) as.dist(1-cor(t(x), method = "spearman"))’).

### Analysis of the human oral metatranscriptomic data set SRP049210

All data sets from the project SRP049210 were downloaded from NCBI SRA database. Quality trimming was performed using CLC Assembly Cell (version 4.0.12) using parameters ‘-f 33 -m 55’ and low-complexity regions of the reads were detected using DUST (from ncbi-blast+2.2.28) with default parameters; reads with >40% of their sequences being masked were subsequently discarded. The remaining reads were then further checked for ribosomal sequences using RIBOPICKER (version 0.4.3) [52]. The nucleotide reads were then translated into peptide reads using FragGeneScan (version 1.17) [23] with parameters ‘-complete = 0 -train = illumina_10’.

Fourteen strains were found important and prevailing across all samples as their genomes recruits the largest amount of reads from all time points (>70%, BWA version 0.7.12 was used for the mapping with default parameters, see ref. [7]). These strains include *Streptococcus salivarius* CCHSS3, *S*. *vestibularis* F0396, *S*. *sp*. C-150, *S*. *mitis* bv_2_str F0392, *S*. *thermophilus* LMD-9, *S*. *parasanguinis* ATCC15912, *S*. *sanguinis* ATCC 49296, *S*. *agalactiae* ATCC 13813, *Veillonella atypica* ACS-134-V-Col7a, *V*. *dispar* ATCC 17748, *Lactobacillus fermentum* IFO3956, *Klebsiella* sp. MS 92–3, *Gemella haemolysans* ATCC 10379 and *Fusobacterium sp*. 2_1_31 [7]. Only the reads that can be mapped onto these 14 genomes were selected for the benchmark experiments. Because the majority of the reads can be mapped onto these 14 strains (64–75%, see S11 Table), the benchmark data set will largely reflect the actual performance of the programs on these human oral MT data sets.

A list of biosynthetic gene cluster profiles registered in antiSMASH2.0 was used as the quires (S3 Table). Ground-truth homologous reads were constructed as by first calling the protein coding regions of these 14 complete genomes using FragGeneScan (using parameter ‘-complete = 1 -train = illumina_10’), searching the querying profiles against the called proteins using HMMER3 and identifying the homologous regions, and then extracting the reads that were mapped to the corresponding genomic intervals of these homologous regions. The ground-truth homologous reads are available online from https://sourceforge.net/projects/hmm-graspx/files/SupplementaryData/OralGroundTruth.tgz. HMM-GRASPx and HMMER3 were run as previously described; and the search results of the programs are available online from https://sourceforge.net/projects/hmm-graspx/files/SupplementaryData/OralSearchResults.tgz.

To perform DE analysis, given a protein family, the reads recruited by HMM-GRASPx/HMMER3 were partitioned according to their BWA mappings against the 14 genomes. The ground-truth read count for a given protein family of a given genome was computed by summing all reads that were mapped to the homologous regions of the corresponding protein family of the corresponding genome. The raw read count generated by HMM-GRASPx and HMMER3 and the ground-truth read count are available from https://sourceforge.net/projects/hmm-graspx/files/SupplementaryData/OralAbundanceCorrelation.tgz. The raw read counts were fed into DESeq2 (version 1.10.1) to perform DE analysis between samples that were collected at 0hr and 6hr. To define condition group in DESeq2, experiments SRX739395, SRX745225, and SRX748266 were pooled as condition 0hr; and experiments SRX748253 and SRX748263 were pooled as condition 6hr. Protein families that were predicted with *P*-values less than 0.05 were considered as differentially expressed. The DE results are available from https://sourceforge.net/projects/hmm-graspx/files/SupplementaryData/OralDEResults.tgz.

For the targeted assembly experiment, all reads from the data sets were searched using HMM-GRASPx and HMMER3. The recruited reads were further assembled in both nucleotide level and protein level. Both protein- and nucleotide-level targeted assembly experiments were performed on 81 antiSMASH2.0 protein families that are longer than 200 HMM states (S8 Table). All corresponding peptide/nucleotide reads from the eight samples whose peptide-products have been recruited by a given program (HMM-GRASPx or HMMER3) were pooled together for assembly.

For protein-level targeted assembly, the peptide reads were assembled using SFA-SPA (version 0.2.1) [21] using default parameters. Short contigs (that are shorter than 60aa, i.e. the expected sum of lengths of two peptide-reads) were discarded. The assembled protein contigs are available online from https://sourceforge.net/projects/hmm-graspx/files/SupplementaryData/OralTargetedAssembly.tgz. The normalized N50 was calculated as the N50 for the contig set over the length of the corresponding querying protein family. Correctness of the contigs was verified by aligning them to the corresponding querying protein family profile using HMMER3 with default parameters. Contigs with a resulting E-value that is more significant than 0.01 was considered as true contigs. The peptide reads were mapped against the contigs using ‘graspx-map’ to estimate the assembly rate.

For nucleotide-level targeted assembly, the nucleotide reads were assembled using SPAdes (version 3.5.0) [18]. Single-end assembly mode was used and no error correction was performed (using ‘—only-assembler’). Short contigs (that are shorter than 180nt, i.e. the expected sum of lengths of two nucleotide-reads) were discarded. The assembled nucleotide contigs are available online from https://sourceforge.net/projects/hmm-graspx/files/SupplementaryData/OralTargetedAssembly.tgz. The normalized N50 was calculated as the N50 for the contig set over the length of the corresponding querying protein family multiplied by three, i.e. the codon length. Correctness of the contigs was verified by first translating them into proteins using FragGeneScan with parameters ‘-complete = 1 -train = illumina_10’ and then aligning them to the corresponding querying protein family profile using HMMER3 with default parameters. Contigs with a resulting E-value that is more significant than 0.01 were considered as true contigs. The nucleotide reads were mapped against the remaining contigs using BWA with default parameters to estimate the assembly rate.

To infer the taxonomic origin of the nucleotide contigs assembled using HMM-GRASPx recruitments, the true nucleotide contigs were further searched against the NCBI non-redundant nucleotide database (NT, downloaded on Oct. 20^th^, 2015) using BLASTN (ncbi-blast+2.2.28) with default parameters and the NCBI non-redundant protein database (NR, downloaded on Oct. 20^th^, 2015) using BLASTX (ncbi-blast+2.2.28). Taxonomy of the contigs that did not hit any NT entries was inferred using KRAKEN (version 0.10.5-beta) [48] with default parameters and RefSeq (downloaded on Jan 13^th^, 2015) as the reference database.

## Supporting Information

S1 Table Taxonomic composition of the simulated marine data set. (XLSX)

S2 Table The Pfam families that were selected as the queries for the benchmark experiment on the simulated marine data set. (XLSX)

S3 Table Benchmark results of HMM-GRASPx, HMMER3, RPS-BLAST, and UProC on a simulated data set where every single genome (the 23 genomes in use is listed in S1 Table) has an even 10X coverage. (XLSX)

S4 Table The Pfam families that were selected as the queries for the benchmark experiment on the human saliva data set SRS013942. (XLSX)

S5 Table The number of true reads (#t.r.) recruited and the number of true contigs (#t.c.) assembled using HMMER3 predicted homologous reads on the human saliva data set SRS013942. (XLSX)

S6 Table The N50 for the contigs that were assembled by SFA-SPA using the HMM-GRASPx-, HMMER3-, RPS-BLAST-, and UProC-predicted homolog reads from the human saliva data set SRS013942. (XLSX)

S7 Table Running time for each program on searching against the simulated marine data set. (XLSX)

S8 Table Running time for each program on searching against the human saliva data set SRS013942. (XLSX)

S9 Table The Pfam families that were selected as the queries for analyzing the AMR profile of the human supragingival and stool metagenomic data sets. (XLSX)

S10 Table Accession numbers for the human supragingival and stool metagenomic data sets that were selected for the AMR protein family profiling experiment. (XLSX)

S11 Table Accession numbers for the human oral metatranscriptomic data set SRP049210 that were selected for the differential expression and targeted assembly experiments. (XLSX)

S12 Table The antiSMASH2.0 profile HMMs that were chosen for the differential expression and targeted assembly experiments on the human oral metatranscriptomic data set SRP049210. (XLSX)

S1 Fig Running time of HMM-GRASPx when searching 303 Pfam protein-family profiles. (PDF)

S2 Fig The two-dimensional hierarchical clustering results generated by using the HMM-GRASPx-predicted AMR families abundance profiles for the human supragingival and stool data sets. (PDF)

S3 Fig The two-dimensional hierarchical clustering results generated by using the HMMER3-predicted AMR families abundance profiles for the human supragingival and stool data sets. (PDF)

S4 Fig Taxonomic profile of the 507 unannotated contigs that were assembled from the in vitro human oral biofilm metatranscriptomic data set through using HMM-GRASPx-based targeted assembly. (PDF)
