# Supplementary material for: Metagenome and Metatranscriptome Analyses Using Protein Family Profiles
Source: PLoS Comput Biol. 2016 Jul 11;12(7):e1004991. doi: 10.1371/journal.pcbi.1004991 (PMC4939949; doi:10.1371/journal.pcbi.1004991)
Supplement: S1 Fig — (PDF) [file pcbi.1004991.s013.pdf]

HMM-GRASPx running time analysis

The asymptotic behavior for the running time of HMM-GRASPx is shown through searching 303 Pfam families (Table S2) against an unbiased simulated data set, in which all protein families have similar abundances. The data set was constructed by randomly selecting 100 protein sequences from Pfam FULL alignment for each family. The selected sequences were subsequently mixed and short peptide reads were randomly generated (with expected length of 32aa, coverage of 10X, and error rate of 1%). The resulting target data set contained 1,512,950 short-peptide reads.

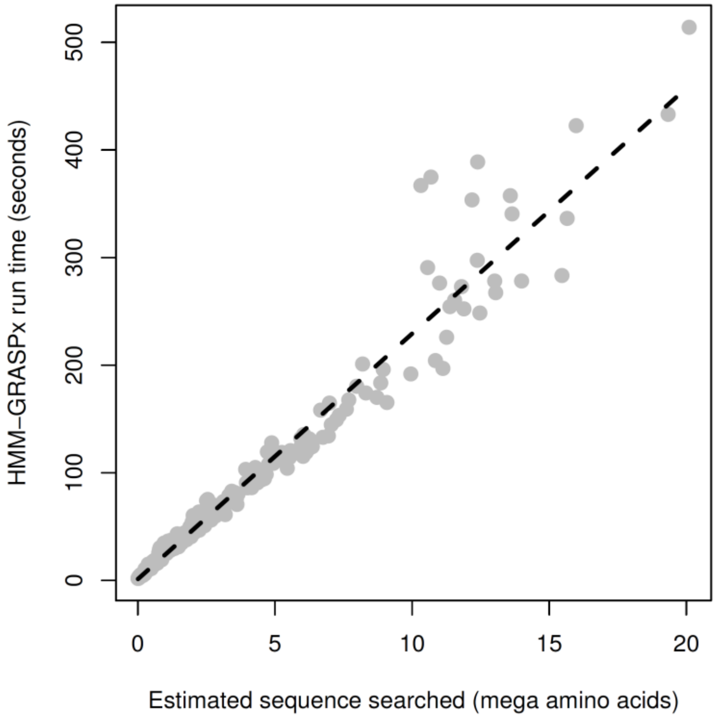

Figure S1: Running time of HMM-GRASPx when searching 303 Pfam protein family profiles against the simulated data set generated as described above. The x-axis indicates the length of the sequence being searched and the y-axis indicates the corresponding running time.

Figure S1 shows that the running time of HMM-GRASPx grows linearly with respect to the total length of the target sequences that have been searched. Such a total length is estimated as the product of the number of identified seeds and the length of the protein family profile.
