# Supplementary material for: Metagenome and Metatranscriptome Analyses Using Protein Family Profiles
Source: PLoS Comput Biol. 2016 Jul 11;12(7):e1004991. doi: 10.1371/journal.pcbi.1004991 (PMC4939949; doi:10.1371/journal.pcbi.1004991)
Supplement: S2 Fig — (PDF) [file pcbi.1004991.s014.pdf]

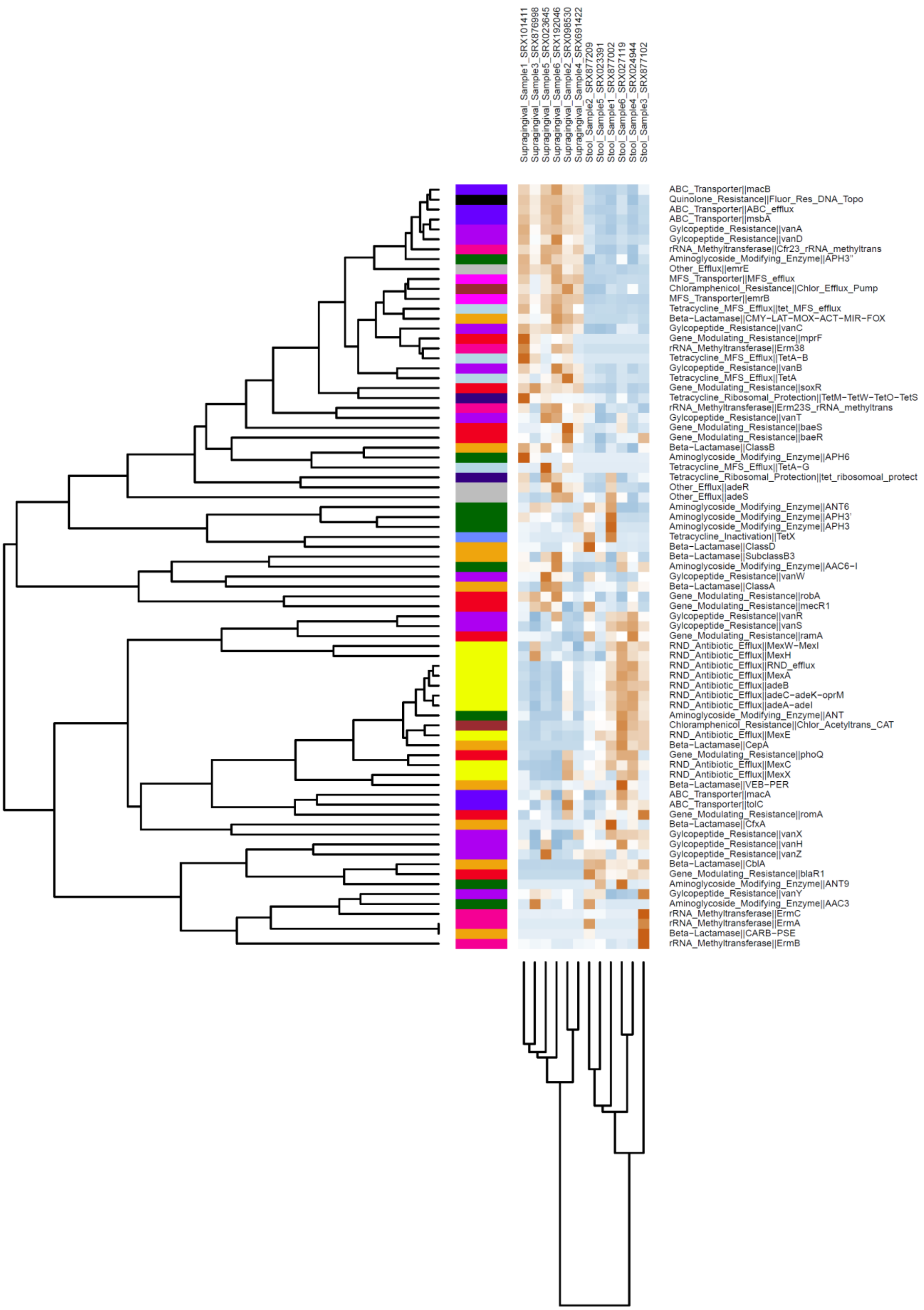

Figure S2: The two-dimensional hierarchical clustering results generated by using the HMM-GRASPx-predicted AMR families abundance profiles for the human supragingival and stool data sets.
