# Supplementary material for: Metagenome and Metatranscriptome Analyses Using Protein Family Profiles
Source: PLoS Comput Biol. 2016 Jul 11;12(7):e1004991. doi: 10.1371/journal.pcbi.1004991 (PMC4939949; doi:10.1371/journal.pcbi.1004991)
Supplement: S4 Fig — (PDF) [file pcbi.1004991.s016.pdf]

Taxonomy inference for unannotated *in vitro* human oral biofilm contigs

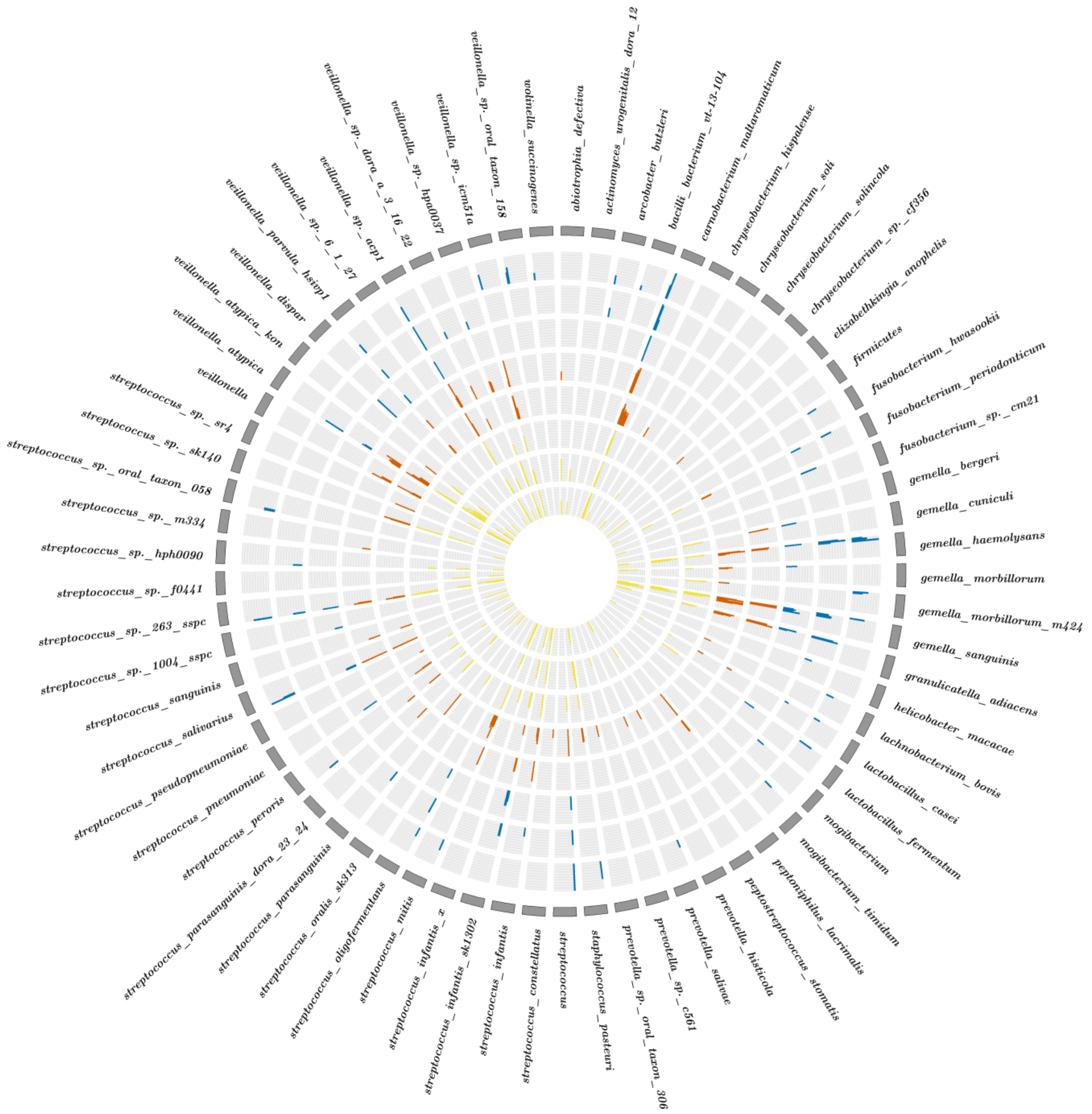

Figure S4: Taxonomic profile of the 507 unannotated contigs that were assembled from the *in vitro* human oral biofilm metatranscriptomic data set through using HMM-GRASPx-based targeted assembly. These contigs lack significant NT hits by using BLASTN (E-value cutoff 10) and thus may originate from uncharacterized species. Taxonomic assignment of these contigs was inferred using their best BLASTX hits against the NR database. Each layer in the plot indicates a data set (blue for 0hr, red for 6hr, and yellow for 9hr). Each bar corresponds to an assembled transcript. Its height corresponds to E-value of the best BLASTX hit (negative logarithm of the E-value, ranging from 0 to100).
